# Managing Commissural Mitral Valve Regurgitation Following Transcatheter Mitral Valve Repair Using the Amplatzer Occluder Device

**DOI:** 10.7759/cureus.78507

**Published:** 2025-02-04

**Authors:** Marc T Zughaib, Harshil Patel, Andrew D Assaf, Souheil Saba, Patrick Alexander

**Affiliations:** 1 Department of Cardiology, Henry Ford Providence Hospital, Southfield, USA

**Keywords:** amplatzer occluder, interventional and structural cardiology, interventional cardiology, mitraclip, mitral regurgitation (mr), structural heart

## Abstract

Mitral regurgitation (MR) is a common valvular abnormality in patients in the Western world. Treatment options include surgery and edge-to-edge repair of the mitral valve leaflets using the MitraClip device. However, MR may recur, and MitraClip can be unsuccessful, posing a challenging management dilemma. We present a case involving a 74-year-old male patient who originally presented due to severe symptomatic MR. The patient originally underwent successful intervention with a MitraClip. However, the patient reported recurrent dyspnea with little effort approximately six months after the index procedure. A repeat 3D transesophageal echocardiogram revealed severe recurrent MR at the medial commissure with an eccentric jet, similar to findings from prior studies. Due to the medial location of the defect, limited space to steer an additional clip, and high surgical risk, the patient was not a suitable candidate for surgical intervention or repeat MitraClip (Abbott Vascular, Santa Clara, CA) placement. The severe recurrent commissural MR was successfully managed using an Amplatzer Patent Foramen Ovale Occluder device (St. Jude Medical, Minneapolis, MN) in an off-label fashion.

## Introduction

Mitral regurgitation (MR) is one of the most common valvular abnormalities found in the elderly population of industrialized nations, with an incidence rate of 10% [1-3]. Untreated patients with MR are at a high risk of developing atrial fibrillation or experiencing worsening heart failure and cardiovascular death. Treatment options for patients with severe MR include surgical mitral valve replacement/repair and minimally invasive mitral valve transcatheter edge-to-edge repair (TEER) with the MitraClip device (Abbott Vascular, Santa Clara, CA) in selected cases. The MitraClip procedure is essentially a procedure where the device is deployed to grasp opposite ends of the mitral valve leaflets, bringing them closer to each other to reduce MR.

The Endovascular Valve Edge-to-Edge Repair Study (EVEREST II) trial demonstrated similar improvement in clinical outcomes and superior safety by percutaneous repair compared with surgical treatment for patients with degenerative MR [4]. While transcatheter repair is safe and effective, the rate of recurrent severe MR could be as high as 10% [3]. Unfortunately, the rates of recurrent MR after TEER are also higher than reported after surgical mitral valve repair [4,5]. Recurrent MR may be located adjacent to the implanted clip or between two clips (intraclip MR), or may be commissural [6]. Several factors play a role in the rate of recurrent MR after TEER. These include a smaller body mass index, a flail leaflet of the mitral valve, a larger number of clips implanted, progressive degeneration of the mitral valve apparatus, and greater residual MR after initial MitraClip placement [7]. The residual MR can lead to significant hemodynamic and clinical outcomes. The residual MR can lead to a sustained volume overload and may contribute to the progression of cardiac remodeling and, subsequently, inadequate leaflet coaptation [8,9].

Options for patients with residual or recurrent MR after a MitraClip procedure include repeat MitraClip placement, surgical valve replacement, or off-label use of medical devices. The procedural success rate of the repeat MitraClip procedure has been reported to be only 62%, compared to >90% for the index MitraClip procedure [10]. In circumstances where valve anatomy is unsuitable for additional MitraClip implantation (e.g., intraclip or commissural MR) and the patient is not a surgical candidate, off-label use of medical devices could be considered.

The Amplatzer Occluder device (St. Jude Medical, Minneapolis, MN), originally designed for closure of atrial septal defects, has been used in an off-label fashion for other conditions, including ventricular septal defect closure, patent ductus arteriosus occlusion, rare congenital anomalies, and, more recently, intraclip or commissural MR [11,12]. The Amplatzer Occluder device is a self-expanding, double-disc device made of nitinol wire mesh. The Amplatzer Occluder device can be a viable option for the treatment of residual post-MitraClip MR, as the placement of additional MitraClips can interfere with the existing clips [5].

We present a case of successful implantation of the device to occlude a residual posteromedial commissural defect responsible for recurrent severe MR after prior successful MitraClip implantation.

## Case presentation

A 74-year-old male patient presented with multiple heart failure admissions within 12 months despite normal left ventricular (LV) systolic function and mild grade I diastolic impairment. His medical history was significant for stable coronary artery disease, hypertension, human immunodeficiency virus, and chronic obstructive lung disease. A transthoracic echocardiogram (TTE) revealed severe eccentric MR with moderate pulmonary hypertension. A 2D transesophageal echocardiogram (TEE) revealed severe degenerative MR directed anterolaterally due to a flail posterior leaflet (P3 scallop). The patient was evaluated for cardiothoracic surgery but deemed a high/prohibitive surgical risk by the multidisciplinary heart team. He agreed to proceed with TEER using MitraClip. The severe 4+ MR was reduced to 1 to 2+ after MitraClip placement along the A3/P3 coaptation point.

Both follow-up TTEs on post-op days 0 and 30 revealed mild-to-moderate residual regurgitation. His effort tolerance improved post-TEER. However, he reported recurrent dyspnea with little effort approximately six months after the procedure. A repeat TEE revealed severe recurrent MR at the medial commissure with an eccentric jet, similar to findings from prior TTE and TEEs. The MitraClip device appeared to be well seated without evidence of leaflet detachment. Despite treatment with optimal medical therapy, the patient remained symptomatic, experiencing dyspnea after mild exertion. A detailed discussion regarding further treatment options was held. These options included surgical repair/replacement, additional MitraClip placement, and closure/sealing of the residual commissural defect. The patient was not a suitable candidate for surgical intervention or repeat MitraClip placement due to the extreme medial location of the defect causing MR, limited space to steer an additional clip, and high surgical risk. After a review of the literature, an off-label treatment with the Amplatzer Occluder device was recommended for severe MR after conservative management had failed.

The procedure was performed similarly to MitraClip-with trans-septal puncture using Baylis (Baylis Medical Company, Mississauga, ON), followed by placing an Agilis steerable sheath (St. Jude Medical, Minneapolis, MN) into the left atrium. The septal defect was crossed successfully with a 260-cm 0.035” Glidewire Advantage (Terumo Medical Corporation, Tokyo, Japan) using both 3D-TEE and fluoroscopic guidance. A 135-cm 0.035” angled NaviCross catheter (Terumo Medical Corporation, Tokyo, Japan) was used to steer the Glidewire Advantage across the aortic valve and into the ascending aorta, followed by advancement of the NaviCross catheter and exchange for Amplatz Super Stiff Guidewire (Boston Scientific, Marlborough, MA). After meticulous measurements via TEE and fluoroscopy, an 18-mm Amplatzer Occluder was chosen to cover the defect and sit securely on both the LV and left atrial (LA) aspects of the valve. This optimally sized device was chosen based on intraoperative TEE measurements of the mitral valve and MR obtained. Additionally, the sizing of the device was guided by the expertise of the Amplatz representative, who was present throughout the case.

**Figure 1:**
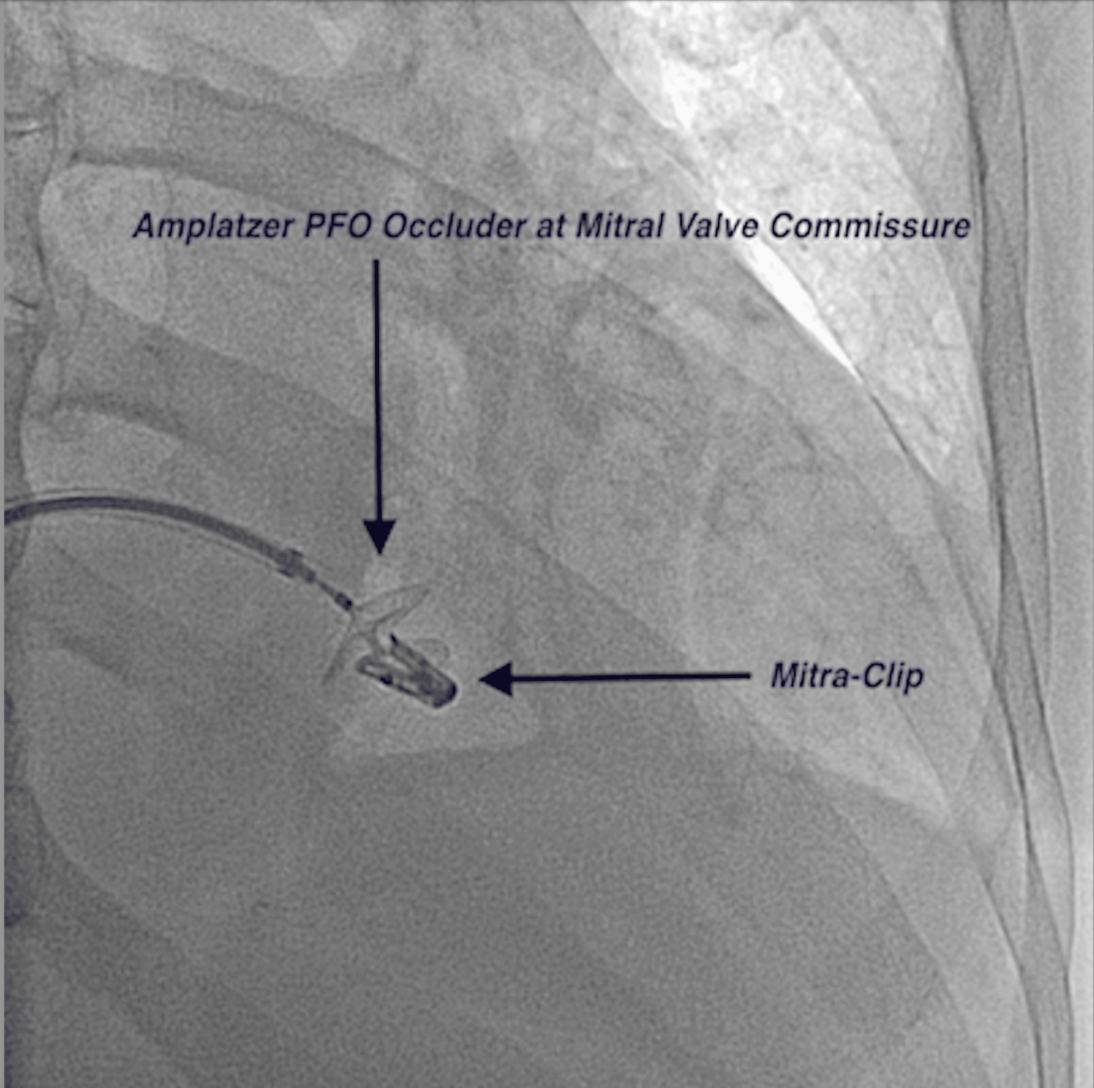
A fluoroscopic view of the Amplatzer PFO occluder device deployed at the mitral valve commissure next to the prior MitraClip PFO: patent foramen ovale

The device delivery sheath was placed into the LV, and the LV disc was deployed. The sheath was then retracted to the ventricular surface of the valve (Figure 1). Fluoroscopic guidance allowed for the appropriate placement of the device.

**Figure 2:**
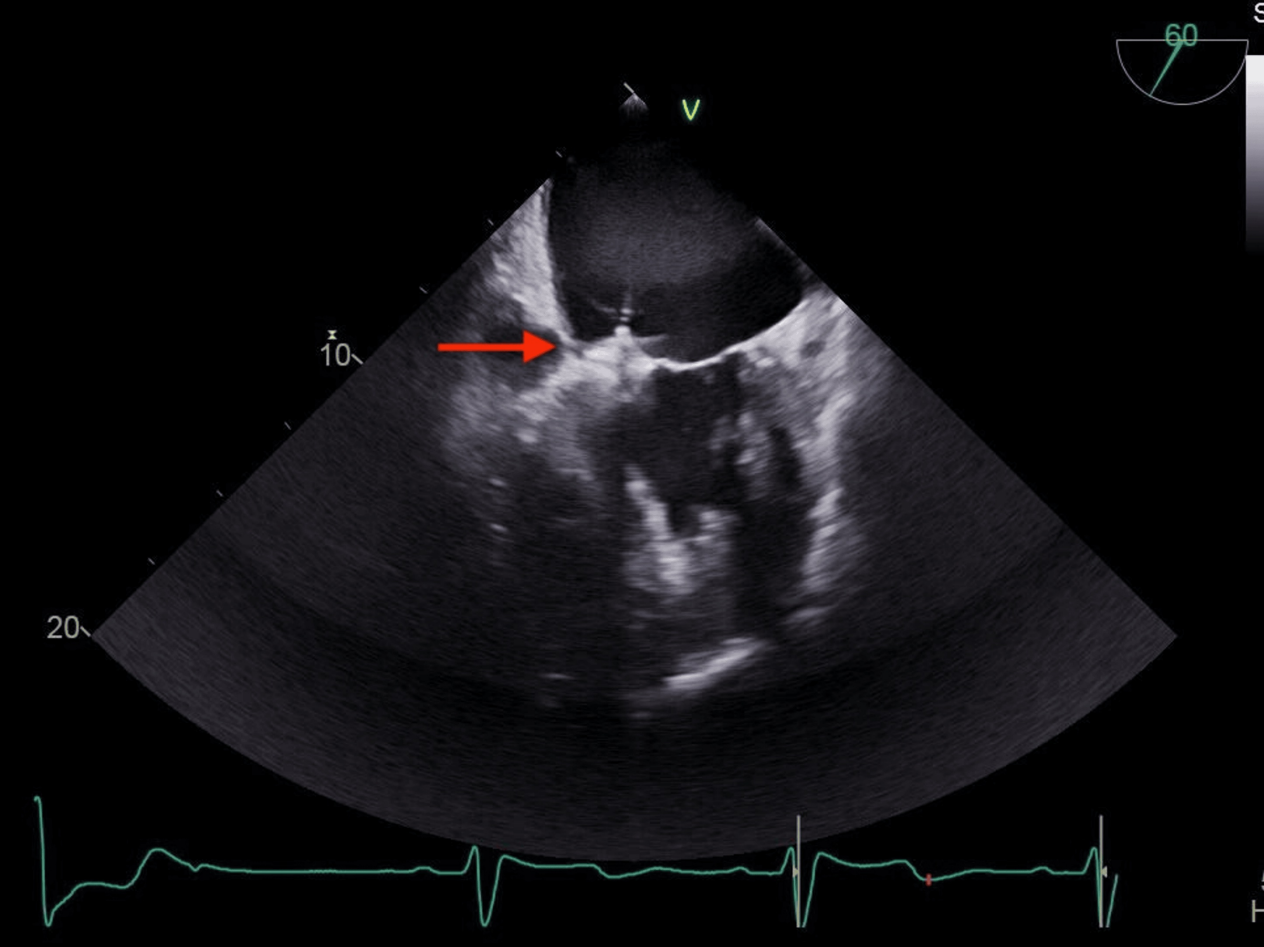
Transesophageal view of the mitral valve at 60°. The Amplatzer device is appropriately seated across the mitral valve (red arrow)

The LA disc was then deployed, followed by a light tug test to ensure firm attachment and stability. Before full deployment, the valve was fully interrogated, evaluating MR reduction, along with the seating and movement of the occluder device and adjacent clip with TEE. The MR was significantly reduced after deployment, with only mild residual regurgitation (Figure 2).

**Figure 3:**
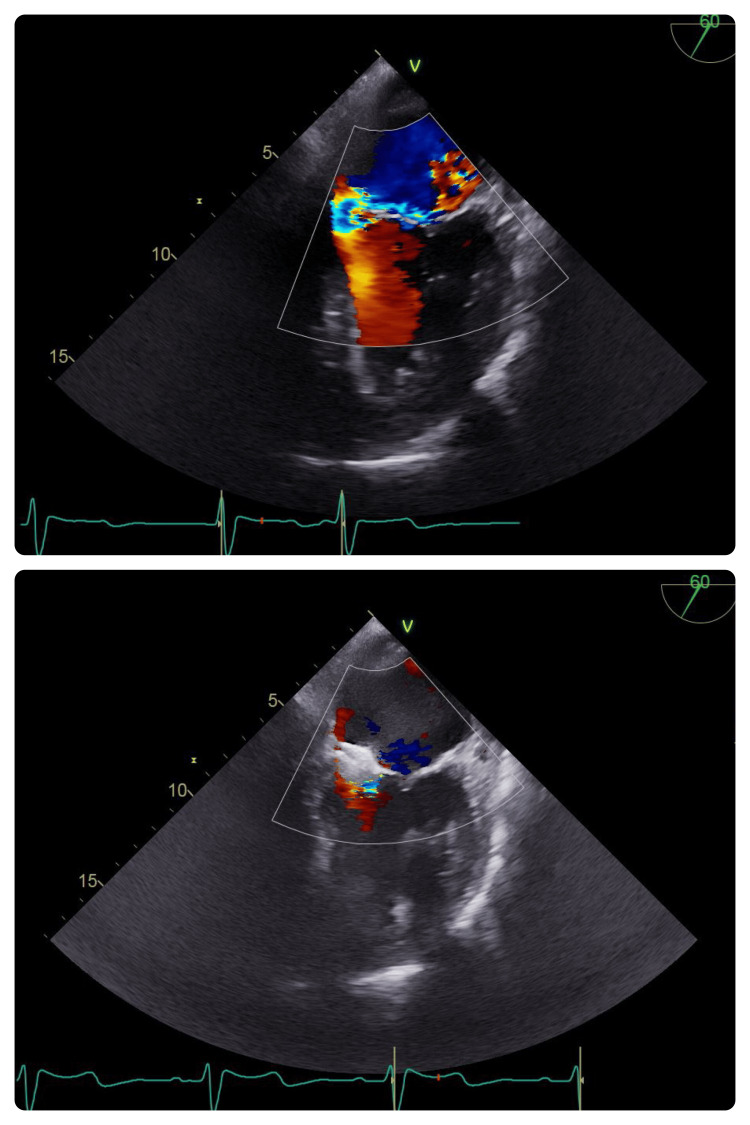
Transesophageal echocardiogram view at 60°. Top: a significant reduction in MR after deployment of the Amplatzer device. Bottom: stable deployment of the Amplatzer device MR: mitral regurgitation

TEE imaging, in conjunction with fluoroscopic guidance, allowed for appropriate device evaluation after deployment. The device appeared stable without excessive motion or significant regurgitation (Figure 3).

The device was then deployed without difficulty. There was no evidence of embolization immediately after deployment or on the postprocedure TTE performed the next day. The postprocedure TTE demonstrated a successful reduction in the MR. The patient was discharged on the following day and closely monitored in the outpatient setting. Repeated echocardiograms were performed at one-month, six-month, and one-year intervals without evidence of recurrent MR. Most importantly, the patient reported significant improvement in their exertional dyspnea.

## Discussion

Over the past decade, the use of MitraClip for TEER has been increasing. This is at least partly due to the results of randomized control trials, such as EVEREST II and Cardiovascular Outcomes Assessment of the MitraClip Percutaneous Therapy (COAPT) [4,12]. The EVEREST II trial compared the clinical outcomes of TEER using MitraClip and conventional mitral valve surgery. The five-year follow-up data demonstrated comparable durability of both strategies, defined as surgery rates, recurrent moderate-to-severe MR, and overall mortality [4]. The COAPT trial compared TEER to guideline-directed medical therapy for managing patients with severe functional MR. The study indicated lower mortality and heart failure hospitalizations in patients who received MitraClip compared with the medical treatment arm at 24 months [13].

While MitraClip implantation is a relatively safe and effective procedure for treating MR, the rate of recurrent severe MR is as high as 9.4% (22 of 234 patients) [3]. The etiologies of recurrent MR after MitraClip implantation are numerous. They include loss of leaflet insertion, leaflet tear at the clip site, partial clip detachment, worsening LV/LA dilatation in cases of functional MR, intraclip MR if more than one clip is used, and worsening degenerative changes in the valve, causing perforation of the valve or iatrogenic damage to the valvular apparatus during the index procedure [3,14].

Recurrent MR can be managed surgically or by transcatheter repair. However, most patients may have a high surgical risk, which may have led them to receive transcatheter therapy the first time. Defects amenable to additional MitraClip placements to correct residual defects may be suitable for reclipping. However, defects can be technically difficult to approach with additional clips but clinically significant enough to warrant correction. In 2016, Taramasso et al. performed an innovative procedure to treat residual intraclip MR using the Amplatzer Vascular Plug II (Abbott Vascular, Santa Clara, CA), which occurred four years after the index TEER [15]. Kubo et al. used the Amplatzer Duct Occluder II to treat residual MR in a series of nine patients after an index MitraClip implantation [6]. Three of these patients had commissural MR as the underlying etiology, similar to our patient. Our patient experienced outcomes similar to those noted in the prior studies, with significant improvement in MR and symptoms/functional status.

The Amplatzer Occluder device, although designed to treat patent foramen ovale, has been used “off-label” to manage other conditions. Baspinar et al. described off-label use of the device in 3.9% of the total number of cases [12]. This device has not been described in the literature for treating recurrent commissural MR. In the present case study, based on the size of the defect and concern for device embolization, a decision was made to use the Amplatzer patent foramen ovale (PFO) occluder rather than the Amplatzer Vascular Plug II. Additionally, the Amplatzer PFO closure device was readily available at our institution, and the operators were very comfortable maneuvering and deploying this device rather than the Vascular Plug II.

Severe MR may lead to heart failure exacerbations, adding significant healthcare expenditure annually, in addition to poor quality of life and shortened life expectancy. The decision to use any medical device in an “off-label” fashion should be backed by the operator’s technical expertise, as well as the availability of necessary resources should any complications occur intraoperatively. Such a decision is best made after an interdisciplinary team composed of structural cardiologists, cardiac imaging specialists, and cardiac surgeons evaluates the case. Our interdisciplinary team ultimately decided that the endovascular approach was more suitable for our patient, but certainly, other institutions may have more expertise or experience with surgical approaches. Future research and studies with off-label use of devices are important as they can provide potential strategies for symptomatic and mortality benefits in patients with limited treatment options. Additionally, these off-label uses of devices may provide insight into the development of specific devices designed to address post-MitraClip recurrent MR.

Our case reiterates the positive impact of correcting a mitral valve defect causing severe MR on the quality of life in patients experiencing worsening symptoms.

## Conclusions

Patients with clinically significant degenerative MR should be offered surgical or minimally invasive options to correct the underlying defect. For patients adjudicated to be poor surgical candidates, MitraClip offers a safe and effective option to correct the underlying defect, with an overwhelmingly high success rate based on five-year outcomes from the Everest II trial. In a minority of these patients presenting with recurrent severe MR due to a residual commissural defect in the mitral valve and overall poor surgical candidates, the Amplatzer Occluder device provided a safe and effective interventional option.
